# Low serum creatinine is associated with type 2 diabetes in morbidly obese women and men: a cross-sectional study

**DOI:** 10.1186/1472-6823-10-6

**Published:** 2010-04-18

**Authors:** Jøran Hjelmesæth, Jo Røislien, Njord Nordstrand, Dag Hofsø, Helle Hager, Anders Hartmann

**Affiliations:** 1 The Morbid Obesity Centre, Vestfold Hospital Trust, Tønsberg, Norway; 2 Department of Biostatistics, Institute of Basic Medical Sciences, University of Oslo, Oslo, Norway; 3 Department of Clinical Chemistry, Vestfold Hospital Trust, Tønsberg, Norway; 4 Department of Medicine, Rikshospitalet University Hospital, University of Oslo, Oslo, Norway

## Abstract

**Background:**

Low skeletal muscle mass is associated with insulin resistance and metabolic syndrome. Serum creatinine may serve as a surrogate marker of muscle mass, and a possible relationship between low serum creatinine and type 2 diabetes has recently been demonstrated. We aimed to validate this finding in a population of Caucasian morbidly obese subjects.

**Methods:**

Cross-sectional study of 1,017 consecutive morbidly obese patients with an estimated glomerular filtration rate >60 ml/min/1.73 m^2^. Logistic regression (univariate and multiple) was used to assess the association between serum creatinine and prevalent type 2 diabetes, including statistically testing for the possibility of non-linearity in the relationship by implementation of Generalized Additive Models (GAM) and piecewise linear regression. Possible confounding variables such as age, family history of diabetes, waist-to-hip ratio, hypertension, current smoking, serum magnesium, albuminuria and insulin resistance (log HOMA-IR) were adjusted for in three separate multiple logistic regression models.

**Results:**

The unadjusted GAM analysis suggested a piecewise linear relationship between serum creatinine and diabetes. Each 1 μmol/l increase in serum creatinine was associated with 6% (95% CI; 3%-8%) and 7% (95% CI; 2%-13%) lower odds of diabetes below serum creatinine levels of 69 and 72 μmol/l in women and men, respectively. Above these breakpoints the serum creatinine concentrations did not reduce the odds further. Adjustments for non-modifiable and modifiable risk factors left the piecewise effect for both women and men largely unchanged. In the fully adjusted model, which includes serum magnesium, albuminuria and log HOMA-IR, the piecewise effect for men was statistically non-significant, but it remained present for women. Patients with creatinine levels below median had approximately 50% (women) and 75% (men) increased odds of diabetes.

**Conclusions:**

Low serum creatinine is a predictor of type 2 diabetes in Caucasian morbidly obese patients, independent of age, gender, family history of diabetes, anthropometric measures, hypertension, and current smoking. Longitudinal studies of both obese and non-obese populations are needed to investigate whether serum creatinine may be causally linked with type 2 diabetes, and if so, precisely how they are linked.

## Background

Obesity and insulin resistance are well established risk factors for type 2 diabetes mellitus (T2DM) [1,2]. Skeletal muscle is the most important site of insulin resistance and accounts for approximately 90% of overall glucose disposal after glucose infusion [3]. Muscle mass has been shown to be inversely associated with insulin resistance [4] and the metabolic syndrome [5]. Conversely, Kuk et al. found that whole-body skeletal muscle mass was not associated with either glucose tolerance or insulin sensitivity in overweight and obese men and women [6]. Creatinine is the only metabolite of creatine which is mainly (98%) located in striated muscle [7], and 24-h urinary creatinine excretion is highly correlated with muscle mass estimates by dual-energy X-ray absorptiometry [8]. Since serum creatinine is highly correlated with 24-h urine excretion (r = 0.82, p < 0.0001) in subjects with normal renal function [9], it may represent an acceptable and easily measured surrogate marker of muscle mass.

*Low serum creatinine *levels were associated with a higher risk of T2DM in a recent study of non-obese middle-aged Japanese men [10], leading the authors to speculate that low creatinine might reflect low muscle mass volume. In addition, glomerular hyperfiltration, which is associated with lower serum creatinine levels, may be associated with increased metabolic risk [11] and future diabetes [12]. Notably, obesity may be considered as a state of relative hyperfiltration, and several lines of evidence indicate that the absolute glomerular filtration rate (GFR) is higher in severely obese subjects than in their lean counterparts [13,14].

We aimed to explore whether serum creatinine is a predictor of T2DM among morbidly obese Caucasian women and men.

## Methods

### Study population, data collection and ethics

The present analysis is based on data from a previously published cross-sectional study of the relationship between PTH and the metabolic syndrome [15]. Briefly, a total of 1,017 consecutive Caucasian morbidly obese patients who attended our tertiary care center between 2005 and 2008 were included in the analysis. All patients had an estimated glomerular filtration rate (eGFR) > 60 ml/min/1.73 m^2 ^[16]. The study was approved by the Regional Committee for Medical Research Ethics (S-05175) and was performed in accordance with the Declaration of Helsinki [17].

T2DM was diagnosed in patients who had a prior history of T2DM or a fasting serum glucose level ≥ 7.0 mmol/l [18]. Homeostasis Model Assessment Insulin Resistance (HOMA- IR) was calculated as ([fasting serum glucose (mmol/l) * fasting serum insulin (pmol/l)]/135) [19]. The log (HOMA-IR) is presented both because it has a stronger linear correlation with glucose clamp estimates of insulin sensitivity and because it is useful for the evaluation of insulin resistance in glucose intolerant individuals, those with mild to moderate diabetes, and those with other insulin-resistant conditions [20].

Lean body weight (LBW) was estimated with gender specific equations which have been validated in extremely obese subjects [21]. LBW (male) = (9270*body weight)/(6680+216*BMI) and LBW (female) = (9270*body weight)/(8780+244*BMI).

The modified Cockcroft-Gault formula (replacing body weight with LBW), which may be appropriate to estimate creatinine clearance in morbidly obese subjects, was implemented in the analysis [22]. It seems superior to the MDRD equation, and it provides a precise and accurate estimate of 24-hour measured creatinine clearance; Creatinine clearance (ml/min) = (140-age)*LBW*serum creatine*1.23*0.85 (if female) [22]. Albuminuria was defined as present if the albumin/creatinine ratio was ≥ 2.5 mg/mmol in men and ≥ 3.5 mg/mmol in women [23].

### Questionnaire physical activity

A sub-group of 495 consecutive patients (recruited between May 07^th ^2007 and September 16^th ^2008) completed a physical activity questionnaire which has been validated by a Norwegian population based epidemiological study [24]. Patients were asked, "How has your leisure-time physical activity been during the last year?" Light activity was defined as no sweating or being out of breath. Hard physical activity was defined as sweating/being out of breath. Duration was classified as hours per week: None, <1, 1-2, 3 and more.

### Laboratory analyses

After an overnight fast blood samples were obtained by venipuncture in vacutainer gel tubes and serum separated from cells within 2 hours. Analyses of serum creatinine, glucose, magnesium and CRP were performed using dry reagent slide technology on the Vitros 950 Analyzer/Vitros FS 5.1 (Ortho-Clinical Diagnostics, New York, USA). The interassay coefficient of variation for creatinine was 2%. Urine-albumin was analyzed using an immunochemical turbidimetric method (Konelab 60i, Thermo Electron Corporation, Helsinki, Finland). Insulin was analyzed in serum using radioimmunoassay (Linco Research Inc, St. Charles, MO). The interassay coefficient of variation for insulin was 8%.

### Statistical analysis

Data are given as mean (standard deviation; SD) or proportions (%) unless otherwise stated. When required, skewed data was log-transformed in order to approximate normality before statistical analyses. Differences between groups were analyzed using independent samples *t*-test for continuous data, whilst χ^2 ^was used for categorical data. Spearman's correlation was calculated to assess bivariate correlations between continuous variables. 95% Confidence Intervals (CI) were constructed using bootstrapping.

Women and men were analysed separately due to the known differences in skeletal muscle mass and serum creatinine.

Logistic regression with predefined explanatory variables was used to assess the odds of T2DM. Expecting non-linearity in the relationship between creatinine and T2DM we also fitted Generalized Additive Models (GAM) [25]. GAM is a natural extension of Generalized Linear Models (GLM), e.g. logistic regression, and allows for all types of functional relationships between dependent and independent variables using splines. As creatinine appeared to be piecewise linear with respect to T2DM in our data, we also fitted piecewise linear logistic regression models for all of the increasingly complex models described below, i.e. estimating the existence and location of a possible breakpoint in the relationship between creatinine and T2DM [26].

We fitted one crude (unadjusted) logistic regression model (model 1) and three separate multiple logistic regression models (models 2-4) for T2DM (yes/no) as the dependent variable. In model 1 serum creatinine as a continuous or dichotomized variable was entered into a logistic regression analysis (no missing values). In model 2 the non-modifiable risk factors age and family history of diabetes were added to model 1 (one missing value). In model 3 the modifiable risk factors; WHR, hypertension and current smoking; were added to model 2 (WHR was substituted for either BMI or lean body weight in supplementary analyses; <1% missing values). Finally, in model 4 other clinically relevant risk factors for T2DM; serum magnesium [27], albuminuria [28,29] and insulin resistance (log [HOMA-IR]); were added to model 3 (8% missing values). A 5% statistical significance level was chosen. The analyses were implemented using SPSS 16.0 (SPSS, Chicago, IL) and R 2.9.0 [30].

## Results

Clinical characteristics and risk factors according to gender and presence or absence of T2DM are shown in table 1 (Additional file 1). Out of the 1,017 consecutive morbidly obese subjects who took part in the study a total of 156 women (23%) and 106 men (32%) had T2DM (p = 0.002 for the difference). When compared to their non-diabetic counterparts the serum creatinine levels of diabetic patients were significantly lower. By contrast, creatinine clearance did not differ significantly between groups.

Serum creatinine had a weak negative correlation with WC (r = -0.08, 95% CI; -0.16 to -0.01), WHR(r = -0.08, 95% CI; -0.16 to -0.02) and BMI (r = -0.08, 95% CI; -0.16 to -0.01) in women, but only with BMI in men (r = -0.11, 95% CI; -0.22 to -0.01). Serum creatinine did not correlate significantly with lean body weight (r = 0.04, 95% CI; -0.04 to 0.12, and -0.01; -0.12 to 0.11, in women and men).

### Serum creatinine (continuous variable) and odds for diabetes

**Figure 1:**
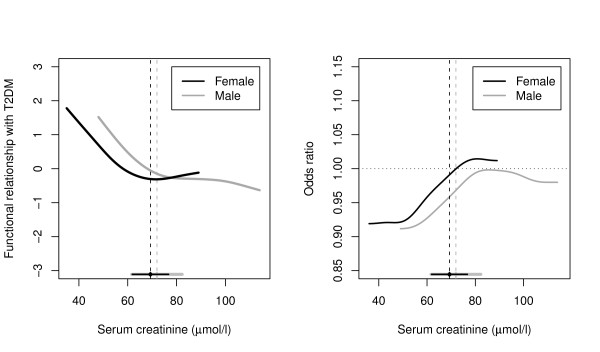
**Relationship between serum creatinine and type 2 diabetes**. Left: Estimated functional relationship between serum creatinine and T2DM using Generalized Additive Models (GAM). Breakpoints and 95% CIs from a piecewise linear models at the bottom. Right: Corresponding odds ratios (approximately 0.90 below and 1.0 above the breakpoints). Given the large uncertainty in the location of the breakpoints the graph does not take the shape of the idealized step function a piecewise linear model assumes, but has a marked transition face.

The unadjusted GAM analysis suggested a piecewise linear relationship between serum creatinine and T2DM (figure 1). Piecewise linear regression models showed that each 1 μmol/l increase in serum creatinine was associated in women with 6% (95% CI; 3%-8%) lower odds of type 2 diabetes below a serum creatinine level of 69 μmol/l, whilst in men it was associated with 7% (95% CI; 2%-13%) lower odds of type 2 diabetes below a serum creatinine level of 72 μmol/l (table 2; Additional file 1). Above these breakpoints the level of serum creatinine did not reduce the odds further. Adjustments for known non-modifiable and modifiable risk factors (models 2-3) left the piecewise effect for both women and men largely unchanged. The results were unaffected by the substitution of WHR for either lean body weight or BMI (data not shown). In the fully adjusted model, which included serum magnesium, albuminuria and insulin resistance (model 4), the piecewise effect for men was statistically non-significant, yet this remained present for women. The effect of serum creatinine was no longer present in men, but among women the inverse relationship between serum creatinine and T2DM remained highly significant before the breakpoint.

Sub-group analysis of data from the 495 patients completing the physical activity questionnaire showed similar results (models 1-3), and the results were not altered by further adjustment for self-reported hard physical activity (data not shown).

### Serum creatinine (dichotomized variable) and odds for diabetes

The breakpoints were located in the proximity of the median serum creatinine for men (73 μmol/l) and women (61 μmol/l). Above these breakpoints the odds of T2DM did not change, whereas the odds increased with decreasing creatinine levels below the breakpoints. For simplification, we therefore also dichotomized creatinine at the medians in order to estimate the average effects of creatinine levels below the breakpoints. Women with creatinine levels below the median had an approximately 50% increase in the odds of T2DM; OR 1.56 (1.09, 2.25) for the unadjusted model and 1.47 (0.93, 2.32) for the fully adjusted model. Men with creatinine levels below the median had an approximately 75% increase in odds of T2DM; OR 1.73 (1.09, 2.79) in the unadjusted model and 1.76 (0.92, 3.39) in the fully adjusted model.

## Discussion

### Statement of principal findings

The results of the present analysis confirm in a population of 1, 017 consecutive morbidly obese subjects with an eGFR > 60 ml/min our initial hypothesis of an inverse association between serum creatinine and T2DM. Each 1 μmol/l increase in serum creatinine was associated in females with 6% (95% CI; 3%-8%) lower odds of T2DM below a creatinine level of 69 μmol/l. In the case of men it was associated with 7% (95% CI; 2%-13%) lower odds of T2DM below a creatinine level of 72 μmol/l. After these breakpoints increasing creatinine levels did not decrease the odds further. Adjustments for known modifiable and non-modifiable risk factors left the piecewise effect for both women and men largely unchanged.

### Interpretation/comparison with other studies

Our findings extend the results from a previous study which demonstrated a significant association between low serum creatinine levels and incident T2DM in Japanese non-obese (mean BMI 23.4 kg/m^2^) middle-aged men (40-55 years) [10], and show this association to be valid for prevalent T2DM in Caucasian morbidly obese women and men between 18 and 75 years of age. In addition, our data indicates that among morbidly obese subjects there is a piecewise linear relationship between serum creatinine and T2DM, with an inverse effect before a breakpoint of approximately 70 μmol/l and no effect above.

Lorenzo et al. recently showed that subjects who had a high GFR (MDRD mean eGFR 121 ml/min/1.73 m^2^) had a two-fold increased adjusted odds, OR 2.29 (1.06-4.97), of incident diabetes as compared with those who had a normal/near normal GFR (mean eGFR 80 ml/min/1.73 m^2^). Although our diabetic patients did not have higher creatinine clearance than their non-diabetic counterparts, they were on average 8 to 9 years older, and creatinine clearance tends to decrease with age. This could explain the apparent absence of association between creatinine clearance and diabetes in the present study, and, thus, we cannot exclude the possibility that glomerular hyperfiltration, which is commonly observed in non-proteinuric T2DM patients [31,32], might partly explain our results.

### Strengths and limitations

The validity of our study is strengthened by the inclusion of a relatively large number of consecutive morbidly obese patients. In addition, the piecewise linear relationship between serum creatinine and T2DM remained stable after adjustments for multiple risk factors. In terms of weaknesses, the cross-sectional design of the study implies that we cannot establish a cause-effect relationship, whilst the exclusion of non-Caucasian participants has meant that we are unable to generalize our results to non-Caucasian populations.

In addition, lean body weight, glomerular filtration rate, and creatinine clearance were estimated rather than measured with gold standard methods [8,16,21,22]. Furthermore, we did not use the oral glucose tolerance test which has a higher sensitivity than fasting blood glucose in the diagnosis of diabetes. However, this is probably not a major problem since fasting glucose has been shown to identify the great majority of morbidly obese patients with unknown diabetes [33]. Finally, the weaker associations and less consistent results among men might be explained by a relatively low number of male participants (type II error).

### Possible explanations

We did not explore possible mechanisms. It might, however, be speculated that low serum creatinine mirrors low muscle mass [7,9], which itself has been associated with insulin resistance [34] and the metabolic syndrome [5], and accordingly provides a higher risk of T2DM. Our study, however, does not support this notion given both that lean body weight, body weight and BMI were not particularly low among patients with low serum creatinine. By contrast, serum creatinine was actually negatively correlated with BMI and not significantly correlated with lean body weight. Finally, adjustments for BMI or lean body weight did not attenuate the inverse relationship between creatinine and T2DM.

### Unanswered questions and future research

Longitudinal studies of both obese and non-obese populations are needed in order to further investigate whether serum creatinine is causally linked to T2DM or whether it rather represents a measure of the disease [35]. Such studies should also explore potential mechanisms and clinical implications.

## Conclusion

Low serum creatinine is a predictor of type 2 diabetes in Caucasian morbidly obese patients independent of age, gender, family history of diabetes, anthropometric measures, hypertension, and current smoking.

## Competing interests

The authors declare that they have no competing interests.

## Authors' contributions

JH contributed to the conception and design, acquisition of data, statistical analysis and interpretation of data, drafted the manuscript and revised it critically for important intellectual content. JR contributed to the statistical analyses, interpretation of data, was involved in drafting the manuscript and revised it critically for important intellectual content. NN contributed to the conception and design, interpretation of data, and revised the manuscript critically for important intellectual content. DH contributed to the conception and design, acquisition of data, interpretation of data, and revised the manuscript critically for important intellectual content. HH contributed to interpretation of data, was involved in drafting the manuscript and revised it critically for important intellectual content. AH contributed to interpretation of data, was involved in drafting the manuscript and revised it critically for important intellectual content. All authors read and approved the final manuscript.

## Pre-publication history

The pre-publication history for this paper can be accessed here:

http://www.biomedcentral.com/1472-6823/10/6/prepub

## Supplementary Material

Additional file 1 **Table 1 and Table 2**. Table 1 and Table 2 Click here for file
