# Supplementary material for: Low serum creatinine is associated with type 2 diabetes in morbidly obese women and men: a cross-sectional study
Source: BMC Endocr Disord. 2010 Apr 18;10:6. doi: 10.1186/1472-6823-10-6 (PMC2861032; doi:10.1186/1472-6823-10-6)
Supplement: Additional file 1 — Table 1 and Table 2. Table 1 and Table 2 [file 1472-6823-10-6-S1.DOC]

**Table 1**. Characteristics of the 1,017 morbidly obese patients according to presence or absence of type 2 diabetes

|  | **WOMEN** | | | **MEN** | | |
| --- | --- | --- | --- | --- | --- | --- |
|  | **Type 2 diabetes** | |  | **Type 2 diabetes** | |  |
|  | **Absent** | **Present** | P-value | **Absent** | **Present** | P-value |
|  |  |  |  |  |  |  |
| No. of patients (%) | 531 (77%) | 156 (23%) |  | 224 (68%) | 106 (32%) |  |
| **Risk factors** |  |  |  |  |  |  |
| Age (years) | 40 (12) | 48 (10) | <0.001 | 41 (11) | 50 (11) | <0.001 |
| Family history of diabetes (%) | 23 | 44 | <0.001 | 20 | 36 | 0.003 |
| Hypertension (%) | 26 | 57 | <0.001 | 38 | 73 | <0.001 |
| Current smoking (%) | 31 | 28 | 0.619 | 18 | 19 | 1.000 |
| **Anthropometry** |  |  |  |  |  |  |
| Weight (kg) | 125 (18) | 124 (20) | 0.686 | 148 (21) | 143 (22) | 0.074 |
| BMI (kg/m^2^) | 44.5 (6.2) | 44.9 (6.6) | 0.525 | 45.5 (5.8) | 44.0 (6.1) | 0.035 |
| Waist-to-hip ratio | 0.95 (0.08) | 0.97 (0.08) | 0.001 | 1.07 (0.08) | 1.10 (0.06) | <0.001 |
| Lean body weight (kg) | 59 (5) | 58 (6) | 0.200 | 83 (7) | 82 (7) | 0.254 |
| **Insulin resistance/magnesium** |  |  |  |  |  |  |
| Log (HOMA-IR) | 0.59 (0.27) | 0.83 (0.33) | <0.001 | 0.69 (0.25) | 0.97 (0.33) | <0.001 |
| Magnesium (mmol/l) | 0.85 (0.07) | 0.81 (0.07) | <0.001 | 0.86 (0.09) | 0.82 (0.08) | <0.001 |
| **Inflammation** |  |  |  |  |  |  |
| CRP (≥ 7 mg/l) (%) | 72 | 74 | 0.622 | 63 | 61 | 0.933 |
| **Renal parameters** |  |  |  |  |  |  |
| Creatinine (μmol/l) | 62 (9) | 59 (10) | 0.001 | 75 (11) | 71 (12) | 0.004 |
| Creatinine clearance (ml/min) | 101 (23) | 97 (24) | 0.091 | 138 (33) | 134 (35) | 0.246 |
| Albuminuria (%) | 6 | 11 | 0.076 | 14 | 27 | 0.008 |
| Microalbuminuria | 6.0 | 9.8 | 0.109 | 10.0 | 15.5 | 0.009 |
| Macroalbuminuria | 0.4 | 1.3 |  | 4.1 | 11.7 |  |

**Table 2.** Odds for type 2 diabetes according to serum creatinine as a continuous variable in piecewise linear logistic regression models.

|  |  |  |  |  | **WOMEN** |  |  |  | **MEN** |  |
| --- | --- | --- | --- | --- | --- | --- | --- | --- | --- | --- |
|  |  |  |  | **Odds ratio**  ***Breakpoint*** | **95% CI*** | **P-value** |  | **Odds ratio**  ***Breakpoint*** | **95% CI*** | **P-value** |
|  |  |  |  |  |  |  |  |  |  |  |
| Model 1 | Unadjusted; serum creatinine only  Before breakpoint  After breakpoint  *Breakpoint* | | | 0.94  1.05  *69* | 0.92-0.97  0.96-1.15  *62-77* | <0.001 |  | 0.93  1.01  *72* | 0.87-0.98  0.97-1.05  *62-82* | 0.015 |
| Model 2 | Model 1 + age and family history of diabetes  Before breakpoint  After breakpoint  *Breakpoint* | | | 0.93  1.07  *71* | 0.90-0.96  0.95-1.20  *64-78* | <0.001 |  | 0.91  1.00  *73* | 0.86-0.97  0.96-1.05  *64-83* | 0.002 |
| Model 3 | Model 2 + WHR, hypertension and current smoking Before breakpoint  After breakpoint  *Breakpoint* | | | 0.94  1.06  *71* | 0.91-0.96  0.94-1.19  *63-79* | <0.001 |  | 0.92  1.01  *74* | 0.86-0.98  0.96-1.07  *64-84* | 0.006 |
| Model 4 | Model 3 + serum magnesium, albuminuria and insulin resistance (log HOMA-IR)  Before breakpoint  After breakpoint  *Breakpoint*** | | | 0.94  1.04  *67* | 0.90-0.97  0.96-1.13  *59-74* | 0.001 |  | 0.98 | 0.95-1.00 | 0.087 |
|  |  |  |  |  |  |  |  |  |  |  |

*CI denotes confidence interval.

Number of missing values: Model 1; none, model 2; 1 woman (<1‰), model 3; 3 women and 4 men (<1%); model 4; 55 women and 27 men (8%)

** in the full model, there was a tendency towards the existence of a breakpoint also for men, but it was not statistically significant.
